# The impact of COVID‐19 control measures on the morbidity of varicella, herpes zoster, rubella and measles in Guangzhou, China

**DOI:** 10.1002/iid3.352

**Published:** 2020-10-07

**Authors:** Di Wu, Qun Liu, Tiantian Wu, Dedong Wang, Jianyun Lu

**Affiliations:** ^1^ Department for Infectious Diseases Control and Prevention Guangzhou Center for Disease Control and Prevention Guangzhou China; ^2^ Pharmacology Laboratory Guangdong Provincial Institute of Biological Products and Materia Medica Guangzhou China; ^3^ Zhongshan School of Medicine Sun Yat‐sen University Guangzhou China

The Coronavirus Disease 2019 (COVID‐19) pandemic have now becoming a worldwide catastrophe, as of September 9, 2020, the conformed COVID‐19 cases are 27,486,960, and 894,983 deaths are reported.^1^ Varicella (chickenpox), rubella and measles are also infectious diseases caused by the respiratory virus. We have noticed that the reported varicella cases, rubella cases, and measles cases in Guangzhou City, China are dramatically decreased during the fight against COVID‐19 pandemic.

We collected the varicella, rubella, and measles data from the China Disease Prevention and Control Information System, which diagnosed by the clinic doctors from 355 medical institutes in Guangzhou City. A total of 88,247 varicella cases, 813 rubella cases and 421 measles cases were included.

There are three peaks of the varicella curve, from the beginning of the year to week 6, and week 16 to 30, and week 40 to the end of the year, and since 2020, the varicella was dramatically decreased when compared to the last 3 years (Figure 1A), which coincides with the two semesters in China. We then analyzed the age‐specific curve of varicella, and found the varicella was most reported at the age of 5, and 52.31% (46,162/88,247) of the cases were under the age of 10 (Figure 1B), also, we found the males were more likely been infected (54.44%, Figure 1C).

**Figure 1:**
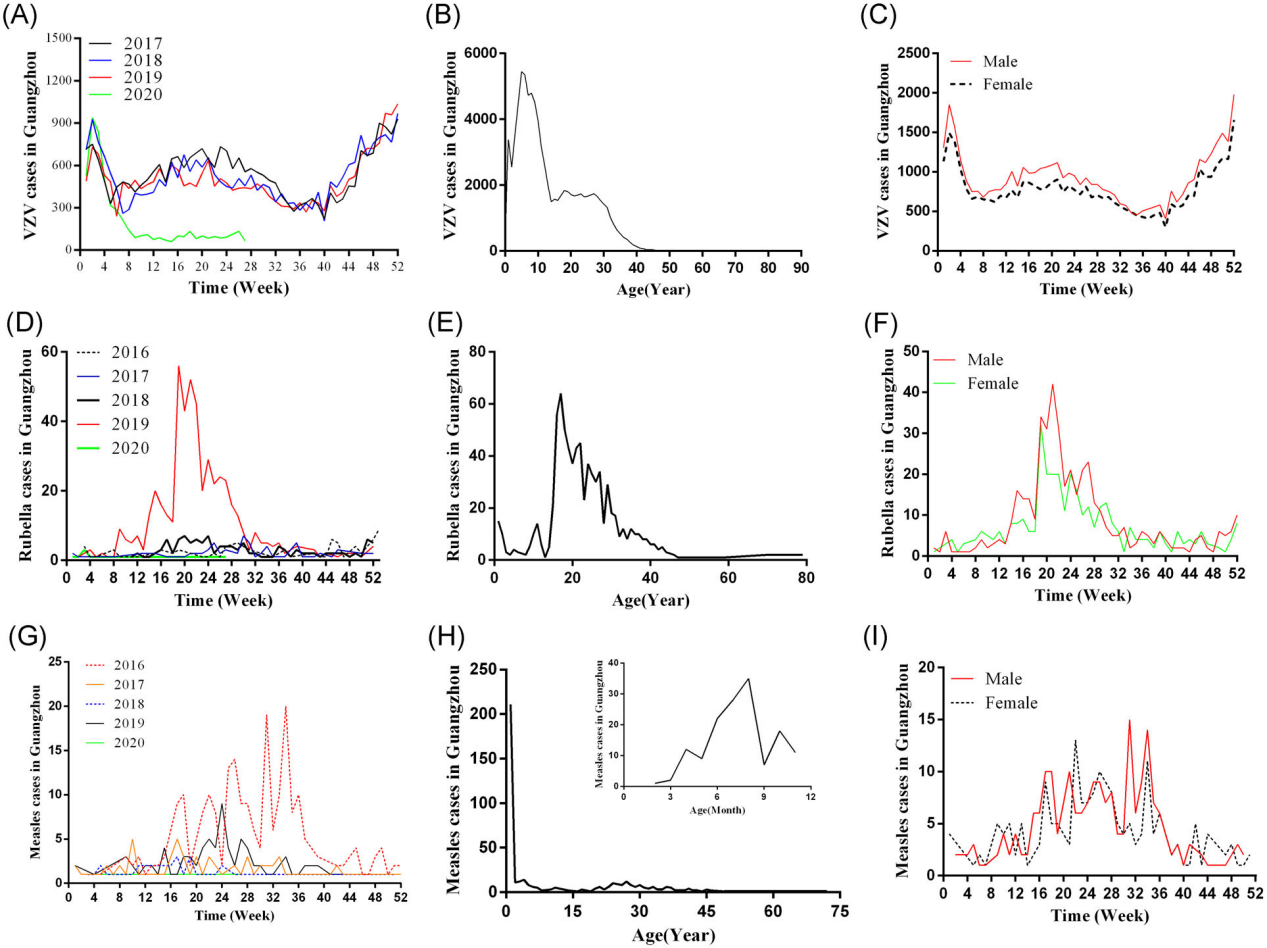
Reduction in VZV, rubella and measles during fight against COVID‐19 pandemic. (A) The weekly reported VZV cases in Guangzhou, China. (B) The age‐specific VZV cases in Guangzhou. (C) The sex‐specific VZV cases by week in Guangzhou. (D) The weekly reported rubella cases in Guangzhou. (E) The age‐specific rubella cases in Guangzhou. (F) The sex‐specific rubella cases by week in Guangzhou. (G) The weekly reported measles cases in Guangzhou. (H) The age‐specific measles cases in Guangzhou. (I) The sex‐specific measles cases by week in Guangzhou

A total of 63.96% (520/813) of the rubella cases was reported in 2019, and since 2020, the varicella was dramatically decreased when compared to the last 4 years (Figure 1D), most cases were reported at the age of 17, and 81.18% (660/813) of the cases were reported between 14 and 37 (Figures 1E), 55.72% (453/813) of the cases are males (Figure 1F).

The measles was at the same situation, there were just four cases was reported in 2020 (Figures 1G), 48.93% (206/421) of the cases were under 1‐year old (Figure 1H), and 52.97% (223/421) were male (Figure 1I).

Studies have reported that the influenza, pneumonia were both dramatically decreased during the fight against the COVID‐19 pandemic,^2^, ^3^, ^4^ the control measures taken to control the SARS‐COV‐2 outbreak also prevented the respiratory infectious diseases such as influenza and pneumonia. Thus, varicella, rubella, and measles are also analyzed and found dramatically decreased since the control measures had been taken to control the pandemic of COVID‐19. Measures such as: (1) the whole population was wearing face masks during they are going outside; (2) keep a social distance; (3) social lockdown and shut down the schools, malls and other places where people congregate; (4) massive temperature measurement; and (5) check and diagnosis the suspect patients with fever. Thus, all respiratory infectious diseases are kept at a lower level of transmission rate when compared to that of the past few years.

Limitation of this study was that the rubella and measles cases are kept a relative lower rate of transmission in the past few years in Guangzhou, and the control measures of COVID‐19 are to some extent changed the behavior when people get sick and go for a doctor.

## CONFLICT OF INTERESTS

The authors declare that there are no conflict of interests.

## AUTHOR CONTRIBUTIONS

Concept and design: Di Wu, Dedong Wang and Jianyun Lu; Acquisition, analysis, or interpretation of data: Tiantian Wu; Drafting of the manuscript: Di Wu and Qun Liu; Statistical analysis: Di Wu; Supervision: Dedong Wang and Jianyun Lu.
